# 17β-Estradiol, a potential ally to alleviate SARS-CoV-2 infection

**DOI:** 10.6061/clinics/2020/e1980

**Published:** 2020-05-18

**Authors:** Ana Cristina Breithaupt-Faloppa, Cristiano de Jesus Correia, Carla Máximo Prado, Roberta Sessa Stilhano, Rodrigo Portes Ureshino, Luiz Felipe Pinho Moreira

**Affiliations:** I Laboratorio de Cirurgia Cardiovascular e Fisiopatologia da Circulacao (LIM-11), Instituto do Coracao (InCor), Faculdade de Medicina FMUSP, Universidade de Sao Paulo, Sao Paulo, SP, BR.; II Instituto de Saude e Sociedade (ISS), Universidade Federal de Sao Paulo (UNIFESP), Santos, SP, BR.; III Faculdade de Ciencias Medicas da Santa Casa de Sao Paulo (FCMSCSP), Sao Paulo, SP, BR.; IV Departamento de Ciencias Biologicas, Universidade Federal de Sao Paulo (UNIFESP), Diadema, SP, BR.; V Laboratorio de Endocrinologia Molecular e Translacional, Escola Paulista de Medicina, Universidade Federal de Sao Paulo (UNIFESP), Sao Paulo, SP, BR.

**Keywords:** 17β-Estradiol, COVID-19, Infection, Coagulation, Inflammation

## Abstract

Considering that female sexual hormones may modulate the inflammatory response and also exhibit direct effects on the cells of the immune system, herein, we intend to discuss the sex differences and the role of estradiol in modulating the lung and systemic inflammatory response, focusing on its possible application as a treatment modality for SARS-CoV-2 patients. COVID-19 patients develop severe hypoxemia early in the course of the disease, which is silent most of the time. Small fibrinous thrombi in pulmonary arterioles and a tumefaction of endothelial were observed in the autopsies of fatal COVID-19 cases. Studies showed that the viral infection induces a vascular process in the lung, which included vasodilation and endothelial dysfunction. Further, the proportions of CD4+ T and CD8+ T lymphocytes were strongly reduced in patients with severe SARS-CoV-2 infection. Estradiol is connected with CD4+ T cell numbers and increases T-reg cell populations, affecting immune responses to infection. It is known that estradiol exerts a protective effect on endothelial function, activating the generation of nitric oxide (NO) via endothelial nitric oxide synthase. Estrogen attenuates the vasoconstrictor response to various stimuli and induces vasodilation in the pulmonary vasculature during stress situations like hypoxia. It exerts a variety of rapid actions, which are initiated after its coupling with membrane receptors, which in turn, may positively modulate vascular responses in pulmonary disease and help to maintain microvascular flow. Direct and indirect mechanisms underlying the effects of estradiol were investigated, and the results point to a possible protective effect of estradiol against COVID-19, indicating that it may be considered as an adjuvant therapeutic element for the treatment of patients affected by the novel coronavirus.

## INTRODUCTION

Severe acute respiratory syndrome coronavirus-2 (SARS-CoV-2), a novel coronavirus, is responsible for the pandemic viral disease outbreak that originated in Wuhan, China, in December 2019. The disease is characterized by severe acute respiratory syndrome, and the virus has already infected more than 3 million people worldwide. No therapies have been shown to be effective to date (1). Data regarding the clinical management of the disease have been published, some of which have demonstrated that there are differences in prevalence and mortality regarding sex (2-4). There is a higher predominance of men affected in several countries (5); in Italy, a prevalence of 70% of men among deceased people was reported (6). Recent data reported by the New York City Department of Health and Mental Hygiene (7) confirmed the higher prevalence and mortality of the disease in men. Moreover, a study of nine pregnant women infected with SARS-CoV-2 showed that the infection did not evolve to severe coronavirus disease 2019 (COVID-19) during the pregnancy (8).

Although there is evidence that the immune response (innate and adaptive) differs between men and women, sexual dimorphism does not receive deserved attention as a potential factor for understanding the different immune responses observed between men and women (9,10). In fact, clinical studies show that women have a lower incidence of pneumonia, sepsis, and multiple organ failure than men (11-13). There are several reports on the participation of female sex hormones in mediating the repercussions caused by trauma followed by hemorrhagic shock (14-23). Important differences exist in epidemiology, pathophysiology, and treatment of cardiovascular diseases, such as coronary artery disease, hypertension, cardiomyopathy, and heart failure. Cardiovascular disorders are also more severe in postmenopausal women (24).

Suba (25) revealed that epidemiological data from SARS and MERS CoV epidemics indicated that the outcome of human coronavirus infections is strongly sex-dependent, suggesting the role of a strong protective estrogen signal in adult female patients compared to age-matched males. In this context, considering that female sexual hormones may modulate the inflammatory response and also exhibit direct effects on the cells of the immune system, here we intend to discuss the sex differences and the role of estradiol in modulating the lung and the systemic inflammatory response and its possible application as a treatment for SARS-CoV-2 patients.

### The effects of SARS-CoV-2

The inflammatory response is a natural defense mechanism of the body to remove harmful stimuli such as pathogens and initiate the recovery process. SARS-CoV-2, the new coronavirus that induces COVID-19, enters the body through the interaction between the S protein on the virus surface and angiotensin-converting enzyme-2 (ACE-2) molecules expressed in epithelial cells in the lungs (26). After SARS-CoV-2 replication in the respiratory and intestinal epithelium cells (27), it can induce an immune response that causes several alterations in the lung including inflammation and respiratory failure. The binding of the COVID-19 protein to ACE-2 has been shown to downregulate its enzymatic activity and decrease angiotensin production (28). This mechanism may be involved in the pathogenesis of pulmonary hypertension and insufficiency caused by SARS-CoV-2 infection (29).

The inflammatory process is accompanied by the activation of the coagulation system, which is characterized by the interaction of coagulation factors, and as observed in severe cases of sepsis, could lead to a disseminated intravascular coagulation process. As recent studies described, COVID-19 is commonly complicated with coagulopathy, and disseminated intravascular coagulation (DIC) may exist in the majority of deaths. In addition to pulmonary pneumonia and inflammation, the presence of vascular dysfunction and thrombosis have been noted in severe COVID-19 patients (30-32).

Because ACE-2, the receptor necessary for virus uptake, is expressed in the cardiovascular system (33), it is expected that this tissue is also susceptible to SARS-CoV-2 infection. Some new studies showed that COVID induces a vascular process in the lung, which includes vasodilation and endothelial dysfunction (34). In this regard, Caruso et al. (35) observed subsegmental vascular enlargement via computer tomography of patients with COVID-19. The presence of thrombosis or acute pulmonary embolism in COVID-19 patients associated with increased respiratory dead space was also reported by Chen et al. (36).

Similar to the disease caused by SARS-CoV identified in 2002, pulmonary involvement is the dominant clinical feature observed in COVID-19. Zhang et al. (30) observed the presence of Rp3 NP SARS-CoV-2 protein in alveolar epithelium cells, including those that were peeled and injured in the alveolar space in lung biopsies. These authors also observed diffuse alveolar damage, epithelial cells peeling with type II reactive hyperplasia of pneumocytes, and fibrinous exudate associated with interstitial fibrosis and chronic inflammatory infiltrate.

Radiological findings in SARS-CoV-2-infected patients have been well-reported in several articles, and the consensus is that COVID-19 induces pneumonia with bilateral lung involvement and ground-glass opacity, the latter being one of the most important radiological characteristic of COVID-19 that can be present even in asymptomatic patients (30,37-39).

According to Mason (40), the pathogenesis of COVID-19 can be divided into three stages. The first asymptomatic phase occurs when the inhaled virus SARS-CoV-2 starts to replicate in the epithelial cells in the nasal cavity. The second phase occurs when the virus migrates through the lung and a more vigorous innate immune response is triggered. The last phase is manifested by hypoxia, ground glass infiltrate, and progression to respiratory failure. A range of 20% to 30% of infected patients will progress to stage 3 of the disease and will develop pulmonary infiltrates that can also evolve into acute respiratory distress syndrome (ARDS). ARDS is an acute inflammatory lung injury, characterized by increased pulmonary vascular permeability, enlarged lung weight, and loss of aerated lung tissue (41). ARDS in its most severe form presents with intense pulmonary inflammation where there is severe hypoxemia (42). In terms of diagnosis, ARDS is characterized by a Pa02/Fi02 ratio equal to or less than 200 mmHg. In essence, the mechanisms underlying the triggering of ARDS are limited to damage to the alveolar epithelium and capillary endothelium (43,44). There is activation of neutrophils and high levels of proinflammatory cytokine release as well as activation of M1-like macrophages and reactive oxygen and nitrogen species. The incidence of ARDS is higher in older patients (45), and aging of the lungs may contribute to the higher mortality rate due to SARS-CoV-2 in elderly and immunosuppressed individuals.

The severe form of COVD-19 is well-characterized by dyspnea, hypoxemia, and pulmonary tissue damage. In addition, 67-85% of critically ill patients admitted to the intensive care unit (ICU) with COVID-19 will develop ARDS, and this will be the cause of death (46,47). Huang et al. (46) reported that dyspnea and lymphopenia was present in more than 50% of infected patients, and all of them showed abnormal findings on a chest CT scan. Considering the inflammatory responses, Lagunas-Rangel et al. (48) suggested that patients with severe COVID-19 show a cytokine storm with acute increased levels of IL-2, IL-7, G-CSF, CXCL10, MCP-1, MIP-1α, TNF-α, and IL-6, similar to patients diagnosed with SARS and MERS. Thus, this suggests that mortality could be due to viral hyperinflammation that exacerbates lung damage.

Some evidence associated high IL-6 levels with the severity and mortality of COVID-19 (49,50), although the levels of interferon (IFN)-γ tend to be somewhat decreased in severe cases compared with moderate cases. These cytokines are also increased in both the serum and bronchoalveolar lavage as well as in lung tissue from ARDS patients (51-53). This alteration in inflammatory response observed in COVID-19 patients could be associated with a reduction in CD4+, CD8+, and NK lymphocytes (36). In this regard, CD4+ T and CD8+ T were strongly reduced in patients with severe COVID-19 compared with those in patients with mild disease (54).

### Sex differences in immune response and inflammation

Steroid sex hormones may affect the strength of immune responses in opposite directions and result in a general difference between males and females, with stronger immune responses in females than in males (for review see Roved et al. (10)). The dominant profile of the immune response may be dependent on hormonal variation during the female sexual cycle. Data indicate that female sex hormones enhance Th2 immune responses and reduce Th1, and this influence has been comprehensively covered in many reviews (9,55,56). Females seems to develop stronger cell-dependent and humoral responses to infection and vaccination. The result of these differences can be observed when comparing the incidence of autoimmune diseases between sexes (57), which is higher in females, and also by the observation of higher incidence of sepsis in males in parallel with higher plasma levels of Th1 cytokines (58-60). Also, the incidence of sepsis in postmenopausal women increases to levels almost equal to those seen in age-matched men (61). In addition to hormonal influences, there is an apparent genetic disparity, since females carry two inherently polymorphic X chromosomes, while males have only one polymorphic X chromosome passed from the mother. The random process of X-chromosome inactivation provides females with a broader panel of proteins and enlarges the potential diversity of cell populations (9).

In women, the CD4+ T cell numbers are higher (62), and the ovarian cycle also influences the T reg cell populations with an increased number when estradiol levels are higher (63). These differences are connected to estradiol influence and affect immune responses to infection. In addition, estradiol exerts anti-inflammatory effects on innate immune responses by reducing monocyte and macrophage inflammatory cytokine release (64), delaying neutrophil apoptosis and enhancing neutrophil annexin-1 expression without increasing their activation (65,66). Experimental data confirm that the leukocyte function in females is more efficient than that in males, detecting and eliminating pathogens more rapidly, and that females have higher numbers of tissue macrophages with a greater density of toll-like receptors; female macrophage phagocytosis is more efficient with NADPH oxidase killing, in parallel with an increased population of resident anti-inflammatory T-lymphocytes (67). The dendritic cell population is also influenced by female sex hormones, with estradiol signaling via estrogen receptor α ER-α to increase the number of new DCs during inflammation (68). In plasmacytoid dendritic cells, which are mostly involved in antiviral responses, estrogen increases the release of type 1 cytokines that are important for an efficient type 1 immune response (69). The action of estradiol via ER-α also promotes activation of type 1 INF-inducible innate pathways, and the IFN pathway genes are highly expressed in females, regulating innate immunity (55).

Considering lung inflammation, mortality in ARDS is higher in males compared to females (70,71). In the respiratory system, sex steroids have been demonstrated to play important roles in the development and maturation of lungs and maintenance of normal lung function (72), and sexual dimorphism impacts the prevalence and incidence of several lung diseases (73). Sex differences were identified in lung physiology and disease and have been studied in animal models. Sex also influences lung development and disease conditions (74). Sex hormones may contribute to the disease pathophysiology or serve as protective factors, depending on the disease involved. Women may be protected from certain age-related biological processes by producing lower levels of reactive oxidant species, important drivers of pathology in age-related pulmonary disease (75). Pulmonary diseases in women are influenced by age because of the variation of their level of estrogen. Increasing age is associated with higher levels of circulating inflammatory mediators and acute phase proteins. The reduction of estrogen, the increased fat tissue, and the presence of subclinical infections contribute to the proinflammatory status of postmenopausal women. The development and progression of age-related diseases is dependent on mechanisms associated with cellular senescence that may be triggered by telomere shortening, which in women occurs at a slower rate than in men (76).

Substantial evidence supports the anti-inflammatory role of systemic estrogens. The mechanisms behind the female protection from infection are reported to be mediated mainly by sex hormones, in particular 17β-estradiol, that can directly influence synthesis and signal transduction of multiple cytokines *in vitro* (67).

### 17β-estradiol therapeutic effects in experimental models

Experimental investigations of several disease models confirm that higher levels of systemic estrogens promote anti-inflammatory responses. 17-β-estradiol is the predominant estrogen during the reproductive years, both in its total serum concentration and in overall estrogenic activity. Estradiol down-regulates immune functions in endothelial cells, including the stimulation of leukocyte adhesion and migration to infected tissues (77-79). It is known that estrogen plays a protective role in endothelial function (80), activating the generation of nitric oxide (NO) via endothelial nitric oxide synthase (eNOS), which is mediated by mechanisms that lead to an increase in NO bioavailability through the induction of gene transcription and activation of eNOS via phosphatidylinositol-3-kinase/serine-threonine kinase (PI3K/AKT) (81). Still, studies by our group using an aortic occlusion model in male rats indicate that previous treatment with estradiol has a beneficial effect on the course of mesenteric ischemia and intestinal injury, preventing mortality due to systemic inflammation (82). Additionally, in an intestinal ischemia and reperfusion (I/R) model, results indicate that female sex hormones, notably estradiol, exert a protective effect on preventing/reducing lung and intestinal injuries caused by systemic inflammation after ischemic trauma (83-85). Proestrus or estradiol-treated females showed reduced lung damage and inflammation in ischemia and reperfusion models (86,87) and in a hemorrhagic shock model (88). In addition, treatment with estradiol showed anti-inflammatory action similar to that of glucocorticoids, with reduced expression of transcription factors involved in the inflammatory response and reduced recruitment of neutrophils by decreasing the production of interleukins, such as IL-8, in addition to chemokines and adhesion molecules (66,89).

Evidence from clinical and experimental studies strongly suggests that estrogens can modulate lung inflammation and allergic reactions, because activation of estrogen receptors modulates immune cells and both innate and adaptive immune responses (55,73). However, the effects of estradiol as a pro or anti-inflammatory factor seem to be dependent on the pathogenesis of the diseases studied. Estradiol plays a critical role in improving outcomes in the settings of trauma, shock, sepsis, myocardial ischemia/reperfusion, and acute lung injury (ALI). Experimental data with an intestinal I/R model show that estradiol treatment in ovariectomized females reduces lung inflammation and exerts these effects by modulating eNOS protein expression in the lungs (83). As permeability edema represents a life-threatening complication of ALI, estradiol effects on the control of lung vascular permeability could be considered among the therapeutic strategies to reduce lung edema (83). In this model, the inflammatory status of the lungs in ovariectomized females remains for a long period after the mesenteric reperfusion has been reestablished and is characterized by the release of proinflammatory mediators and danger signals. The treatment with estradiol is able to downregulate the lung inflammation and its capacity for releasing cytokines (90). In males, using the same I/R model, estradiol treatment was effective in reducing lung inflammation even at 1h after the reestablishment of intestinal perfusion (84). Thus, the effects of estradiol are important in the modulation of an already established lung inflammatory response. It is noteworthy to point out that the proposed treatment was acute and could be considered a clinical alternative.

Estrogen attenuates the vasoconstrictor response to various stimuli and induces vasodilation in the pulmonary vasculature during stress situations like hypoxia (91). This is mediated by increased levels of prostacyclin and NO as well as decreased levels of endothelin-1 (ET-1). It is well-established that estradiol exerts important vasoactive effects by activation of eNOS, as well as modulation of vasoactive substances released from endothelial cells or the direct vasodilation effect via relaxation of smooth muscle cells (92). The vasoactive effects of ET-1 can be influenced by estradiol and are organ-dependent (93), and estradiol and its metabolites inhibit ET-1 synthesis (94). Ovariectomized female rats present higher ET-1, and estradiol replacement reduces ET-1 peptide expression (95). Thus, estradiol acts as a protective vasoactive agent against deleterious microcirculatory conditions, such as brain death and ischemia/reperfusion injury (82,96). Brain-dead rats treated with 17β-estradiol exhibit reduced lung injury, mainly because of its actions on endothelial and inducible nitric oxide synthase (iNOS) (96). Regarding injury, estrogen can inhibit vascular responses and thus prevent proatherosclerotic events (97). The maintenance of organ homeostasis depends on a complex network of systems, and reduction of blood flow may have profound consequences on organ status. Microcirculatory function following trauma can be affected by sex hormones (98), altering tissue perfusion and influencing inflammatory processes. Estrogen exerts a variety of rapid actions, initiated after coupling with membrane receptors, which may positively modulate vascular responses in pulmonary disease and help to maintain microvascular flow.

### COVID-19, coagulation, and estradiol influence

Although one of the consequences of severe COVID-19 is the development of ARDS, some studies indicated that respiratory phenotypes induced by COVID-19 are slightly different from those classical phenotypes observed in ARDS patients (99). COVID-19 patients developed a severe hypoxemia early in the course of the disease, which is silent most of the time (100). Most recently, small fibrinous thrombi in small pulmonary arterioles and a tumefaction of endothelial were observed in autopsies in cases of fatal COVID-19 (101). This could indicate the activation of the coagulation cascade in these patients. In addition, they also reported diffuse alveolar damage with intense epithelial viral cytopathic effects both in the alveolar and small airways. More interestingly, the presence of thrombus occurred in both damaged and preserved lung parenchyma areas. All together, these features suggested that anticoagulant drugs could have beneficial effects in COVID-19 patients.

Estrogen reduces platelet aggregation, the opposite effect of testosterone (102,103). Notelovitz et al. (104) studied the short-term effects of estrogens on the coagulation-fibrinolysis process in surgically treated women, and the results did not show abnormal or thrombogenic changes in coagulation parameters. *In vitro*, 17β-estradiol is able to inhibit platelet aggregation by promoting Ca^2+^ extrusion or reuptake activity, and its action is dependent on the increase of NO synthesis (105). Platelet reactivity plays a pivotal role in thrombus formation, and *in vitro* data suggest that although women have a higher magnitude of platelet reactivity, their response to aspirin is similar or even larger compared to that of men (106). Experimental data show that a chronic high physiologic level of estrogen equivalent to that observed in pregnant mice had a significant inhibitory effect on platelet aggregation (107). Estrogen mediates beneficial effects on the cerebral microcirculation and moderated cerebral thrombotic mechanisms by enhancing the plasma levels of NO (108). Moreover, estrogens exert positive effects on the pulmonary vasculature by increasing prostacyclin release and NO production by eNOS (109). Cowman et al. (110) evidenced a connection between age and decreases in platelet tracks, platelet translocation, and unstable platelet interactions. The altered platelet function associated with aging was more profound in females compared with that of males and could be a result of a reduction in estrogen levels in women.

### The role of estrogen and estrogen-related compounds in antiviral mechanism activity

Considering virus-induced lung inflammation, the excessive inflammation leads to tissue damage. In this regard, T cells seems to be reduced in COVID-19 patients, and the present T cells seemed to be functionally exhausted (111). Severe COVID-19 can induce ARDS in more than 50% of patients, and neutrophils are considered to be the central cells involved in the pathogenesis of ARDS. Infected female mice that were administered estradiol showed pulmonary recruitment of neutrophils and virus-specific CD8 T cells, releasing more INF-γ and TNF-α (112). Moreover, exogenous estrogen treatment in female mice infected with H1N1 reduced total pulmonary inflammation and the levels of pro-inflammatory genes in the lung (113).

Many viruses are affected by estrogen at the molecular level, specifically in the replication machinery and maturation of the virion, but to date, no evidence has been presented for SARS-CoV-2. Despite the well-known role of estrogen in inflammation to combat pathogens and infections, it has been shown that estrogen receptors participate in repressing transcription virus genes. One example is the hepatitis B virus (HBV) infection, where ER-α interferes with virus gene expression through (HNF)-4α (hepatocyte nuclear factor 4α) binding to HBV enhancer I in hepatocytes (114). In the hepatitis C virus (HCV) infection model *in vitro* (Huh7 cells), 17β-estradiol reduced virus infection by interfering with assembly and/or virus release, disrupting the virus life cycle, and this effect was inhibited by anti-estrogen Fulvestrant (115). Moreover, HCV-infected women, after postmenopause, exhibit a reduced response to antiviral therapy and greater incidence of hepatic fibrosis and hepatic carcinoma (116,117).

Nevertheless, the most likely antiviral effects of estrogen are mediated by inflammation modulation. Regarding the cells that participate in the immune response, dendritic cells play a crucial role. Escribese et al. (118) infected dendritic cells with Newcastle disease virus (NDV) and treated the cells with 17β-estradiol. The authors observed a diminished type I IFN synthesis, also reducing the CD4 T cell response, suggesting that estrogen has an antiviral activity on RNA viruses.

According to the evidence that female sex hormones exert antiviral activity in many organs, the use of selective estrogen receptor modulators (SERMs) has been extensively explored in experimental systems to reduce virus infections (reviewed by Montoya & Krysan, (119)). In general, SERMs have been used for several therapeutic purposes, such as in the treatment for infertility and osteoporosis, but are mainly related to cancer as adjuvants in chemotherapy, such as tamoxifen. In viral infection, this compound was suggested to be considered as an antiviral therapy, because of its properties of disrupting viral replication during HIV (human immunodeficiency virus) infection in monocytes and CD4+ T-lymphocyte cell lines (120). Tamoxifen has also displayed a capacity to inhibit HCV replication via estrogen receptor assembly to RNA-dependent RNA polymerase NS5B (121,122). Murakami et al. (122) showed that various SERMs reduce the production of HCV RNA, preferentially related to the extracellular but also intracellular virus proteins, suggesting that SERMs may be a multi-targeted drug that interferes with several processes, comprising virus entry and replication.

Of note, the most approximate model of SARS-CoV-2 viral infection is found in MERS-CoV and SARS-CoV. Both have been demonstrated to be affected by toremifene citrate (a classical SERM), which delayed virus infection due to the inhibition of the late endosome virus trafficking (123), and a similar effect was observed previously with Ebola virus infection (124). In addition, both *in vitro* and *in vivo* experiments showed that toremifene and clomifene present antiviral activity against a large variety of Ebola virus strains (125,126). Importantly, it was also demonstrated that SERMs can elevate the survival rate of infected animals by 90% and 50% when using clomifene and toremifene, respectively. Mechanistically, it has been proposed that toremifen could target envelope proteins GP1 and GP2, which permit the viral attachment and access to host cells, decreasing its stability and resulting in reduced endolysosomal membrane fusion (127). This may be considered good evidence to support that estrogen and estrogen-related compounds play a major role in antiviral therapies for SARS-CoV-2.

## CONCLUSION

**Figure 1:**
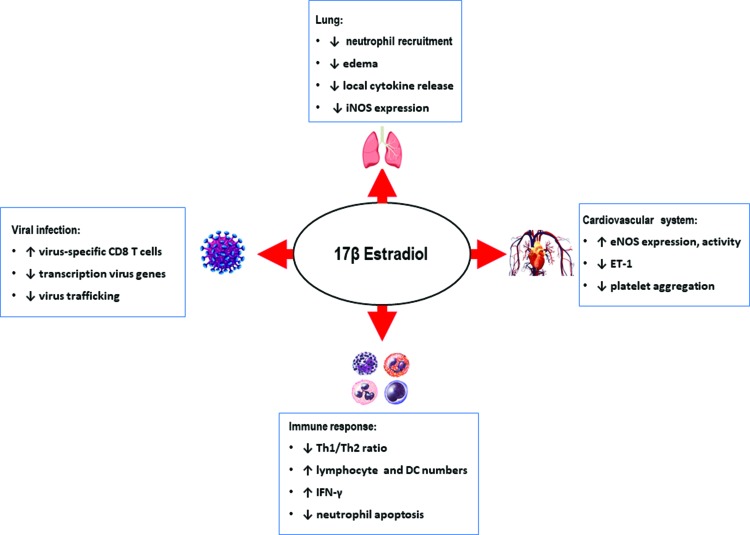
Effects of 17β-estradiol in different compartments/systems.

Abundant literature highlights sex differences in immune response and its influence on the incidence and severity of diseases. Considering trauma, shock, and infection, the female sex is associated with advantageous outcomes. Since the hormonal context exerts an important influence on the homeostasis control and the defense mechanisms, it is important to consider the beneficial effects of estrogens with regard to providing better control of defense and the different immune cells involved, in addition to cardiovascular system protection and flow maintenance. Direct and indirect mechanisms underlying the effects of estradiol were investigated, and the results point to a possible positive effect of estradiol as an adjuvant therapeutic element for the treatment of patients affected by the novel coronavirus, SARS-CoV-2 (Figure 1). Studies are being carried out in order to analyze the effects estradiol on SARS-CoV-2 *in vitro*, and in parallel, there is already a clinical trial in course.

## AUTHOR CONTRIBUTIONS

All of the authors conceived the study and were responsible for the manuscript drafting, editing, and review.
